# From spent alkaline batteries to Zn_*x*_Mn_3−*x*_O_4_ by a hydrometallurgical route: synthesis and characterization

**DOI:** 10.1039/c8ra06789a

**Published:** 2018-09-28

**Authors:** Lorena Alcaraz Romo, Ana López-Fernández, Irene García-Díaz, Paloma Fernández, Ana Urbieta, Félix A. López

**Affiliations:** Centro Nacional de Investigaciones Metalúrgicas (CENIM-CSIC) Avda. Gregorio del Amo 8 28040 Madrid Spain f.lopez@csic.es +34-915-347425 +34-915-538-900; Facultad de Ciencias Físicas, Universidad Complutense de Madrid, Ciudad Universitaria Plaza de las Ciencias 28040 Madrid Spain

## Abstract

A series of Zn/Mn binary oxides with different molar ratios (1.4–11) were synthesized *via* co-precipitation from a solution obtained through the acidic (HCl) leaching of a black mass originating from the mechanical recycling of spent alkaline and Zn–C batteries. The oxides obtained were characterized by X-ray diffraction (XRD), Fourier-transform infrared spectroscopy (FTIR), X-ray photoelectron spectroscopy (XPS), and Raman spectroscopy. Magnetic properties of the samples were also investigated. The Raman spectroscopy results showed all the binary metallic oxides belong to the Zn_*x*_Mn_3−*x*_O_4_ (0.25 ≤ *x* ≥ 1.75) type. All showed a spinel crystalline structure. The saturation magnetization decreases with the Zn/Mn molar ratio; a maximum of 13.19 emu g^−1^ was found for the molar ratio of 11 at the Curie temperature (25.5 K). XPS showed that all the synthesized compounds contained Mn^2+^, Mn^3+^ and Mn^4+^. Mn^2+^ was the most prominent at a molar ratio of 11, Mn^3+^ was most common at a molar ratio of 2, and Mn^4+^ at 1.4.

## Abstract 2

A series of Zn/Mn binary oxides with different molar ratios were synthesized *via* co-precipitation from a solution obtained through the leaching of a black mass originating from the mechanical recycling of spent alkaline and Zn–C batteries.

## Graphical abstract

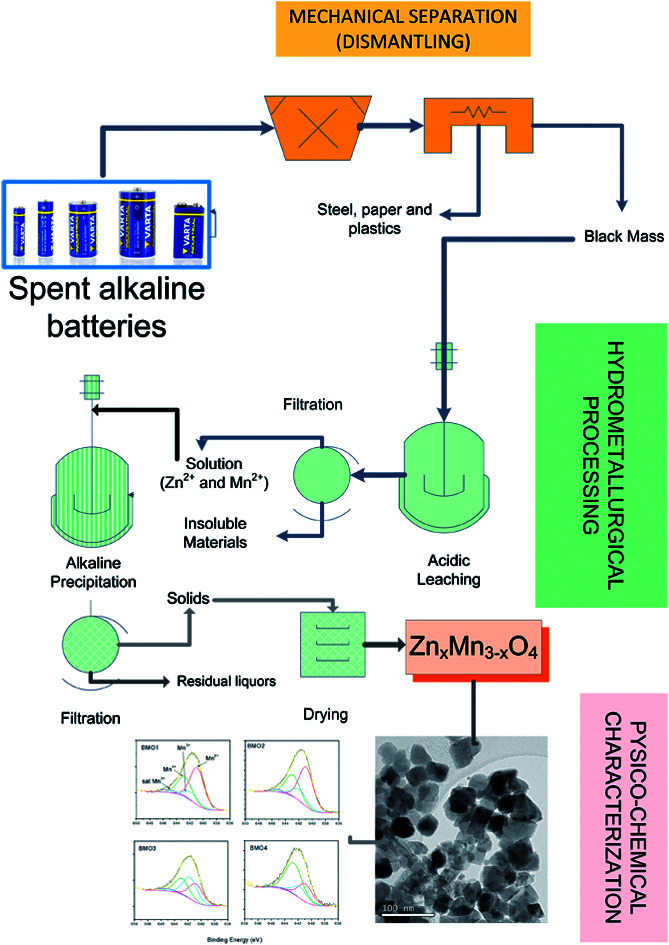

## Introduction

In recent decades, technological advances have allowed batteries to be used as energy sources in many electronic devices (toys, computers, cell phones, watches, remote controls, cameras, *etc.*).^1^ Unfortunately this has led to the need to dispose of increased quantities of spent batteries.^2^ The yearly global demand for batteries is currently growing at 7.7% – by 2019, battery consumption will reach 80 000 million units per year, representing a market worth US$120 billion.^3^ In Spain, only about 24% of spent batteries (representing some 3031 tonnes) are currently collected for proper disposal. By 2019, however, it is expected that 65% will be recovered for recycling.^4^

The main components of batteries are manganese dioxide (positive electrode), zinc (negative electrode), an electrolyte (KOH or ZnCl_2_ + NH_4_Cl), and the steel casing. These compounds and any other heavy metals they may contain (*e.g.*, cadmium, mercury, lead, lithium) may seep out, negatively affecting the environment and human health.^4–6^ These dangers, plus the elevated costs associated with the adequate management of these spent products, make the recycling of batteries an attractive option.^7^

The techniques developed for processing spent batteries fall into three groups: mechanical separation, pyrometallurgical treatment, and hydrometallurgical treatment.^8^ Mechanical separation is commonly required before any further processing, especially hydrometallurgical processing. The aim is to separate the electrodes, steel casing and any plastic or paper components.^9^ Mechanical separation usually involves cutting/milling, magnetic separation, dimensional separation (screening), eddy current separation (ECS) employing Foucault currents, and the final milling of the particulate fraction.^7^ The result is a so-called 'black mass' (consisting largely of electrolytes, graphite, and oxides of zinc and manganese). Although pyrometallurgical treatment is the most commonly used method^10,11^ hydrometallurgical treatment is gaining attention given its low cost and more environmentally friendly nature.^12^

The hydrometallurgical treatment of spent battery black mass takes place in several stages: pre-treatment, acidic or alkaline leaching, and the recovery of the Zn and Mn *via* electrolysis, liquid–liquid extraction, or selective precipitation.^13^ The use of biological processes for this is also of interest since they are likely to be less environmentally harmful.^14,15^ Several hydrometallurgical processes have been patented for the industrial scale recovery of Mn and Zn from spent batteries, including the Bateaux,^16^ Recupyl^17^ and Revabat^18^ processes, among others.

Previous work performed by the present authors showed it is possible to obtain highly pure ZnO from black mass *via* leaching with ammoniacal ammonium carbonate.^19,20^ This ZnO has good luminescent properties and can be used in gas sensors.^21^

Ternary compounds of metallic oxides that contain transition metals of the AB_2_O_4_ type are currently a focus of research. In this context ZnO, doped with a certain amount of Mn, forms interesting spinel structures of potential use in shortwave magneto-optical^22^ and spintronic devices.^23^ In addition, semiconductors doped with small quantities of transition metals (TM), known as diluted magnetic semiconductors (DMS), are of great technological interest given their capacity to control spin as well as electric charge, a property that makes them potentially useful in the manufacture of new generation spintronic products.^24^

In addition, ZnO and TM doped ZnO compounds (such as Cr, Mn, V) exhibit photocatalytic applications under UV and solar radiation.^25,26^ ZnO : Mn also processes the capacity to capture CO_2_ and SO_2_.^27^ All of this makes these compounds potentially useful in several technological applications.

A number of studies have reported various ways of synthesizing the spinel ZnMn_2_O_4_. Courtel *et al.* synthesized ZnMn_2_O_4_ nanoparticles from their corresponding acetates *via* a hydrothermal process^28^ and *via* coprecipitation.^29^ The production of ZnMn_2_O_4_ microspheres *via* solvothermal synthesis from the corresponding acetates has been also reported.^30^ Recent studies have also shown that ZnMn_2_O_4_ spinel can be obtained *via* autocombustion processes^31,32^ and sol–gel methods,^33,34^ among others. However, while several authors have described the recovery of different valuable metals from spent lithium ion batteries,^35–38^ few of them describe in detail a comparative study of the structural characteristics and magnetic properties of these type of compounds.

The present work describes the synthesis of Zn_*x*_Mn_3−*x*_O_4_ (0.25 ≤ *x* ≥ 1.25) compounds with molar ratios ranging between 1.4 and 11, and with a spinel crystalline structure, *via* coprecipitation from the solution obtained following the acidic leaching of spent battery black mass.^35–38^

## Experimental procedure

### Preparation of black mass

Black mass produced from spent alkaline and Zn–C batteries was provided by Envirobat España, S.A (Guadalajara, Spain). Table 1 shows its mean chemical composition, as revealed by X-ray fluorescence (XRF) analysis using a PANalytical Axios wavelength dispersive spectrometer (4 kW). The mineralogical composition was determined by X-ray diffraction (XRD) using a Siemens D5000 diffractometer equipped with a Cu anode (Cu Kα radiation) and a LiF monochromator. Morphological analysis was performed by scanning electron microscopy (SEM) using a FEI Inspect microscope equipped with an X-Ray energy dispersive spectrometer (EDS).

**Table 1:** Chemical composition of the starting black mass (wt%)

Compound	Black mass
Na_2_O	7.75
MgO	0.23
Al_2_O_3_	0.46
SiO_2_	1.69
P_2_O_5_	0.99
SO_3_	0.64
Cl	1.76
K_2_O	6.70
CaO	0.36
TiO_2_	0.191
MnO	43.30
Fe_2_O_3_	1.42
Co_3_O_4_	0.03
NiO	0.28
CuO	0.08
ZnO	26.88
SrO	0.04
ZrO_2_	0.01
Nb_2_O_5_	0.01
CdO	0.01
SnO_2_	0.03
BaO	0.13
La_2_O_3_	0.05
CeO_2_	0.10
PbO	0.04
C_total_	8.20
S	0.24

### Synthesis of zinc and manganese oxides

Different binary metallic oxides (BMO) with different proportions of Zn/Mn were produced from the black mass *via* (1) the acidic (HCl) leaching of Zn and Mn, followed by (2) the precipitation of Zn^2+^ and Mn^2+^ cations in an alkaline medium.

### Acidic leaching

100, 200, 300 or 400 g of black mass were dispersed in a 1 L suspension in 250 mL milliQ water plus 500 mL of 6 M HCl (Panreac®) and 250 mL of H_2_O_2_ (Panreac®). After mixing (500 rpm) for 1 h at room temperature, the suspension was filtered through a Millipore Holder filter at a pressure of 7 bars. The solid phase was discarded and the resulting filtered solutions termed L_i*q*_, where *q* refers to the 100, 200, 300 or 400 g used. The Zn and Mn contents of these solutions were determined by atomic absorption spectroscopy (AAS) using a Varian Spectra AA 200 spectrometer. The pH of the solutions was, in all cases, approximately 0. The solutions were then subjected to precipitation in an alkaline medium.

### Precipitation in an alkaline medium

**Fig. 1:**
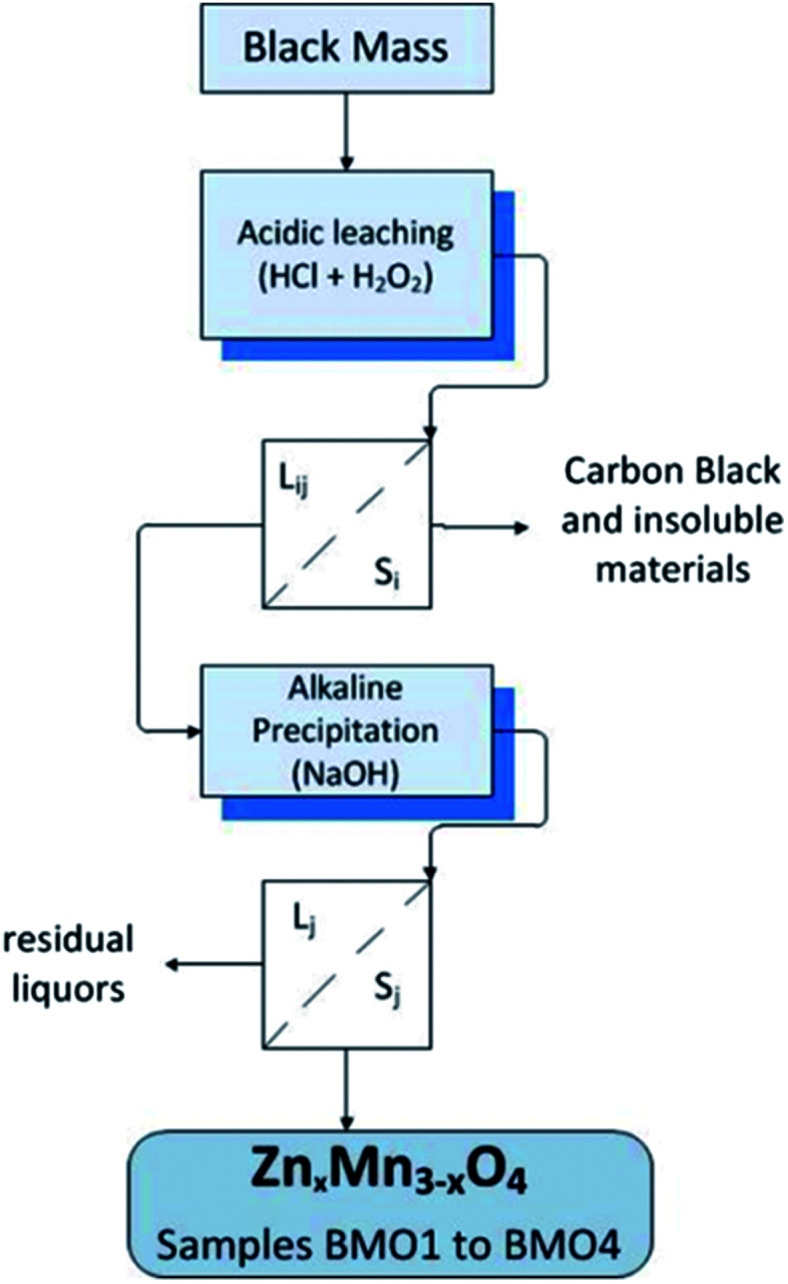
Synthesis routes followed for the production of the binary metallic oxides.

The filtered solutions (L_i*q*_) were subjected to a precipitation procedure involving 6 M NaOH. The pH was monitored using a pH meter at room temperature until a value between 12–14 was obtained – allowing the precipitation of zinc and manganese oxides. The solutions were then filtered, producing a solid phase of different hues of brown containing the Zn/Mn binary oxides (BMOs) and a final liquid phase (L_j_) that was discarded. The solids obtained were termed BMO1, BMO2, BMO3 and BMO4 with reference to their precipitation from L1 to L4 respectively. Fig. 1 summarizes the procedure for the production of these BMOs.

### Characterization of the Zn/Mn binary metallic oxides

The chemical composition of the Zn/Mn BMOs was determined by XRF using the above-mentioned PANalytical Axios wavelength dispersive spectrometer. Their mineralogical composition was determined by XRD using the above-mentioned Siemens D5000 diffractometer. The Rietveld method was used to calculate structural parameters from the XRD patterns, using TOPAS v4.2 software (Bruker ASX) and taking into account the crystallographic information for the different phases from Pearson's crystal structure database for inorganic compounds.^39^ The morphology of all the BMO samples was studied by scanning electron microscopy (FE-SEM) using a JEOL JSM 7600 apparatus, as well as by transmission electron microscopy (TEM) using a JEM 2100 HT device.

Fourier-transformed infrared spectroscopy (FTIR) using a Varian 670 FTIR spectrometer (spectral range 1600–400 cm^−1^, spectral resolution of 4 cm^−1^) in transmittance mode was also performed. In addition, micro-Raman spectra were obtained using a confocal Horiba Jovin-Yvon LabRAM HR800 system. The samples were excited by a 633 nm He–Ne laser on an Olympus BX 41 confocal microscope with a 10× objective.

The temperature dependence of the magnetic susceptibility was measured in the 2–300 K range in a magnetic field of 1000 Oe using a Quantum Design XL-SQUID magnetometer. Hysteresis measurements were taken at 10 K with a maximum field of 5 T.

The surface chemistry of the BMO samples was examined by XPS. Spectra were recorded using a Fisons MT500 spectrometer equipped with a hemispherical electron analyzer (CLAM2) and a non-monochromatic Mg Kα X-Ray source operated at 300 W. Spectra were collected at a pass energy of 20 eV (typical for high-resolution conditions). The area under each peak was calculated after subtraction of the S-shaped background and fitting the experimental curve to a combination of Lorentzian and Gaussian lines of variable proportions. Binding energies were calibrated to the C 1s peak at 285.0 eV. The atomic ratios were computed from the peak intensity ratios and reported atomic sensitivity factors.^40^

## Results and discussion

### Characterization of the black mass

**Fig. 2:**
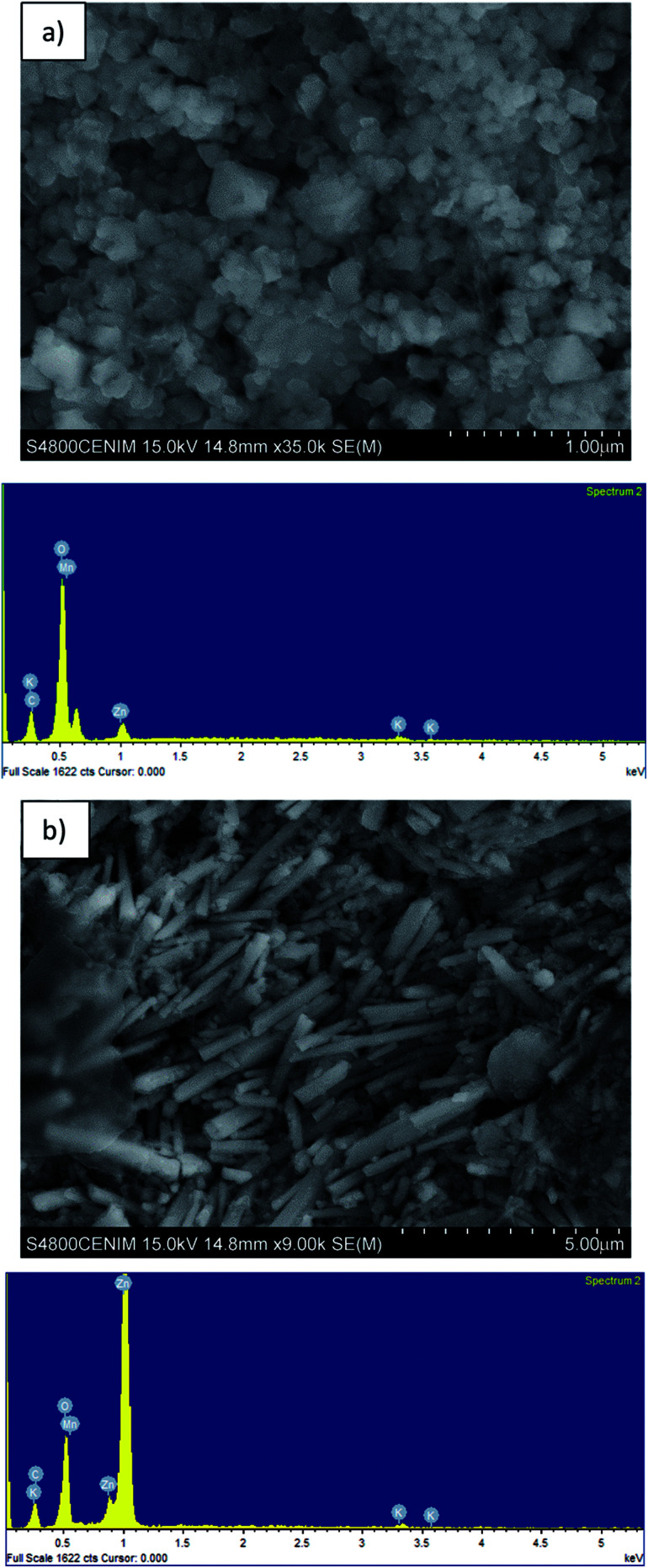
SEM and EDS images of the: (a) hetaerolite, and (b) zinc oxide present in the black mass.

Table 1 shows the composition of the black mass, which consisted mainly of Mn (36.8 wt%) and Zn (23.7 wt%). The major crystalline phases were zincite (ZnO), hetaerolite (ZnMn_2_O_4_) and sylvite (KCl). Fig. 2 shows SEM images and the corresponding EDS microanalysis which reveal that the hetaerolite (Fig. 2a) and zincite (Fig. 2b) phases are present.

### Synthesis of binary metallic oxides

Table 2 shows the composition of the acidic leachates. These solutions were pinkish in colour, characteristic of solutions containing Mn^2+^ ions, the intensity of the colour depending on the Mn^2+^ concentration. Eqn (1) and (2) show the dissolution reactions:^12^

1 ZnO (s) + 2H^+^ → Zn^2+^ (aq) + H_2_O (aq)

2 ZnMn_2_O_4_ (s) + 6H^+^ + H_2_O_2_ → Zn^2+^ (aq) + 2Mn^2+^ (aq) + 4H_2_O (aq) + O_2_ (g)

**Table 2:** Zn and Mn content (g L^−1^) obtained from AAS and lixiviation efficiency (%)

Sample (acid leaching)	Element		Lixiviation yield (%)	
	Zn	Mn	Zn	Mn
L_1_	24.8	88.8	81.0	99.8
L_2_	35.9	116.9	77.9	88.9
L_3_	48.6	101.6	79.4	60.0
L_4_	63.0	63.2	73.0	47.0

Table 2 reveals how the Mn content decreases as the amount of dissolved black mass increases. The highest Mn content was obtained for L_i200_ sample. The concentration of Zn increases with the amount of black mass dissolved.

During the alkaline precipitation process, the precipitation of Zn(OH)_2_ (eqn (3)) starts at pH 7.5 and increases with the pH. The precipitate was white.

3 Zn^2+^ (aq) + OH^−^ (aq) → Zn(OH)_2_ (s)

Zn(OH)_2_ (*K*_sp_ at 25 °C = 3 × 10^−16^) continues to form until a pH of 11.5 is reached, after which (bearing in mind the amphoteric nature of this compound) it dissolves to form zincate ions (eqn (4)):

4 Zn(OH)_2_ (s) + OH^−^ (l) → ZnO_2_^2−^ (aq) + 2H_2_O (aq)

The Mn^2+^ is stable up to pH 8.5, after which manganese hydroxide (*K*_ps_ = 2.5 × 10^−13^) is formed, along with mixed Zn/Mn hydrates (eqn (5)):

5 *x*ZnO_2_^2−^ (aq) + (3−*x*)Mn^2+^ (aq) + 2*x*OH^−^ (aq) → Zn_*x*_Mn_3−*x*_(OH)_2*x*_ (s)

These hydrated oxides are rapidly oxidized to form anhydrous oxides with a spinel structure (eqn (6)):

6 Zn_*x*_Mn_3−*x*_(OH)_2*x*_ (s) + 2O_2_ (g) → Zn_*x*_Mn_3−*x*_O_4_ (s) + 2*x*H_2_O (aq)

Depending on the Zn and Mn contents of each solution, the obtained value of *x* is different, ranging from 0.25 to 1.2. When *x* = 1, the product is ZnMn_2_O_4_ (sample BMO3). XRF analysis of BMO3 reveals a Zn content of 21.6 wt% and a Mn content of 49.6 wt%, similar to the stoichiometric values obtained (27.3 wt% Zn and 45.9 wt% Mn).

Ameri *et al.*^41^ proposed a similar mechanism for the formation of ZnMn_2_O_4_*via* the electrodeposition of Zn^2+^ and Mn^2+^ solutions obtained from the dissolution of zinc and manganese nitrates. In their work, the initial solutions possessed an excess of Mn^2+^ in relation to the stoichiometry of ZnMn_2_O_4_ and therefore they obtained nanostructured ZnMn_2_O_4_/Mn_3_O_4_. Song *et al.*^42^ proposed a similar mechanism for the formation of ZnMn_2_O_4_*via* solvothermal synthesis dissolving zinc and manganese nitrates solutions in citric acid and cetyltrimethyl ammonium bromide (CTAB).

The reactions shown above explain the formation of the Zn/Mn BMOs. In the case of BMO4 large amounts of ZnO were detected. This is a consequence of the high concentration of Zn^2+^ in the acidic solution (63.0 g L^−1^); during the alkaline precipitation process some Zn^2+^ did not combine with the Mn^2+^. This excess Zn^2+^ precipitated out as Zn(OH)_2_, and later became ZnO through dehydration.

Regarding the composition, with the exception of the odd minority elements, all are composed of Zn and Mn, the exact content of each depends on the amount of solution subjected to alkaline precipitation The Zn and Mn contents and stoichiometry as determined by XRF (Table 3) and Rietveld refined XRD (Table 4) respectively agrees quite well for samples BMO1, BMO2 and BMO3. The contents Zn and Mn for BMO1 (22.8 wt% and 49.2 wt%) agrees with the stoichiometric proportion for the Zn_0.85_Mn_2.15_0_4_ phase (23.4 wt% and 49.7 wt% respectively). In the case of BMO2, the Zn and Mn contents are 8.1 wt% and 66.2 wt% respectively, which agrees with the stoichiometric proportion for the Zn_0.25_Mn_2.75_O_4_ phase (7.1 wt% and 65.3 wt% respectively). In the case of BMO3, the agreement is also good, the Zn and Mn contents of BMO3 are 26.7 wt% and 46.3 wt% respectively, while the stoichiometry of the ZnMn_2_O_4_ phase is 27.3 wt% Zn and 45.9 wt% Mn. However, a discrepancy seems to appear for the Zn and Mn contents of BMO4, since according to stoichiometry (Zn_1.25_Mn_1.75_O4) the expected values for the contents of both elements are 29.5 wt% and 36.5 wt% respectively, while from the XRF data, the Zn content is 40.2 wt% (Table 3). Nevertheless, if we take into account the data from Table 4, in this sample, an equivalent amount of ZnO (12% wt) is detected, then accounting for the 10.7 wt% excess of Zn observed.

**Table 3:** XRF results for the metal binary oxides powder (expressed in %, wt/wt oxides) obtained after alkaline precipitation and extraction efficiency (%)

Sample	Content (wt%)		Extraction efficiency (%)	
	Zn	Mn	Zn	Mn
BMO1	22.8	49.2	19.2	64.3
BMO2	8.1	66.2	16.8	86.2
BMO3	26.7	46.3	46.7	72.0
BMO4	40.2	36.0	82.3	48.0

**Table 4:** Mineralogical composition and calculated cell parameters of Mn/Zn binary oxides from the Rietveld refinement

Sample	Mineralogical composition (%)	Cell parameters of samples	
		*a* (Å)	*c* (Å)
BMO1	Zn_0.85_Mn_2.21_0_4_ (95%)	5.756	9.266
	Impurities (5%)	—	—
BMO2	Zn_0.25_Mn_2.75_0_4_ (95%)	5.766	9.393
	Impurities (5%)	—	—
BMO3	ZnMn_2_0_4_ (96%)	5.758	9.240
		—	—
BMO4	Zn_1.25_Mn_1.75_0_4_ (72%)		
	ZnO (12%)	5.789	8.976
	Impurities (16%)	—	—

### Characterization of the binary metallic oxides

**Fig. 3:**
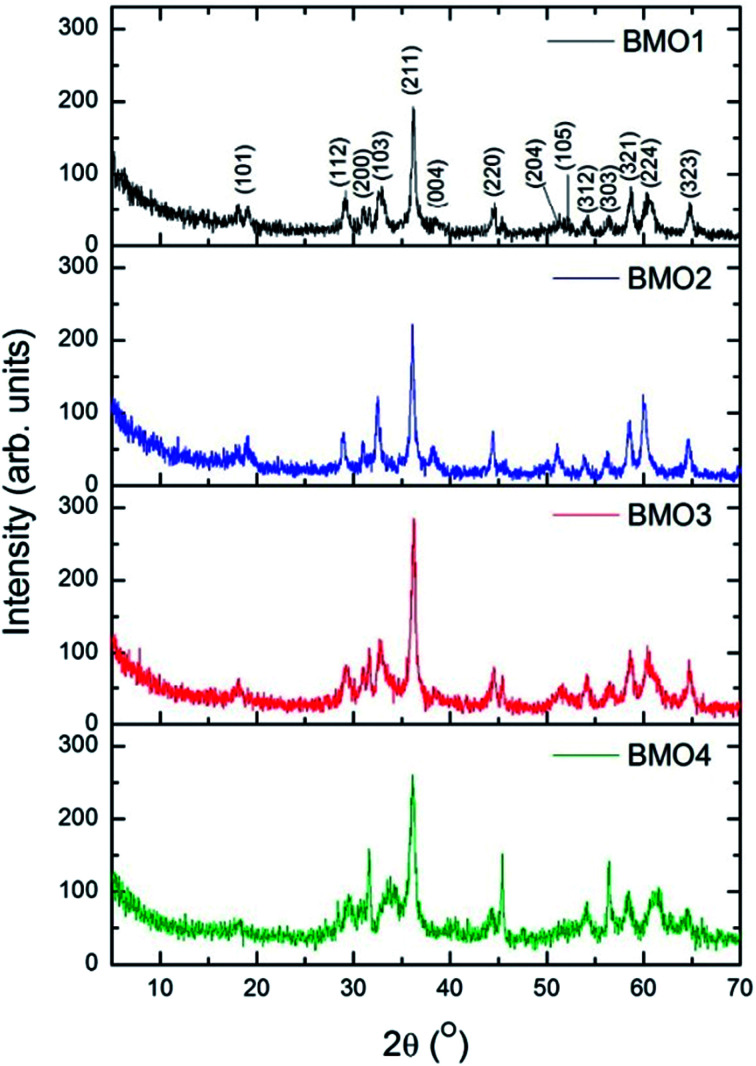
X-ray diffractograms for the synthesized binary metallic oxides.

The composition of the crystalline phases was examined by XRD. Fig. 3 shows the diffractograms recorded. In most cases, a tetragonal symmetry with a *I*4_1_/*amd* spatial group are obtained, which, according to JCPDS Card no. 24-1133, fits well with an spinel-type structure. However, the diffractogram for BMO4 also shows a diffraction maximum corresponding to ZnO (JCPDS Card no. 36-1451).

Table 4 shows the composition of the crystalline phases of the BMOs as determined by Rietveld refinement; where a very clear variation in the proportions of Zn and Mn is observed. The different Zn/Mn BMOs obtained also show a very high purity (95–96%).

The measured value for the a lattice parameter of the ZnMn_2_O_4_ phase is 5.758 Å, *i.e.*, slightly higher than the values reported in the literature (5.709–5.722 Å). The value for *b* parameter (9.240 Å), however, is much closer to previously reported values (9.222–9.238 Å).^37,41,42^ The value of a parameter for the Zn/Mn BMO stoichiometries decreases with increasing Zn content. This agrees with previous results reported for ZnMn_2_O_4_ phases obtained by the ceramic method.^43^

The intensities of the diffraction maxima decrease as the Mn content increases. An increasing Mn content is therefore associated with a reduction in the crystallinity of the BMOs. Other authors have reported similar results for ZnO doped with Mn obtained *via* hydrothermal and sol–gel synthesis.^44,45^

**Fig. 4:**
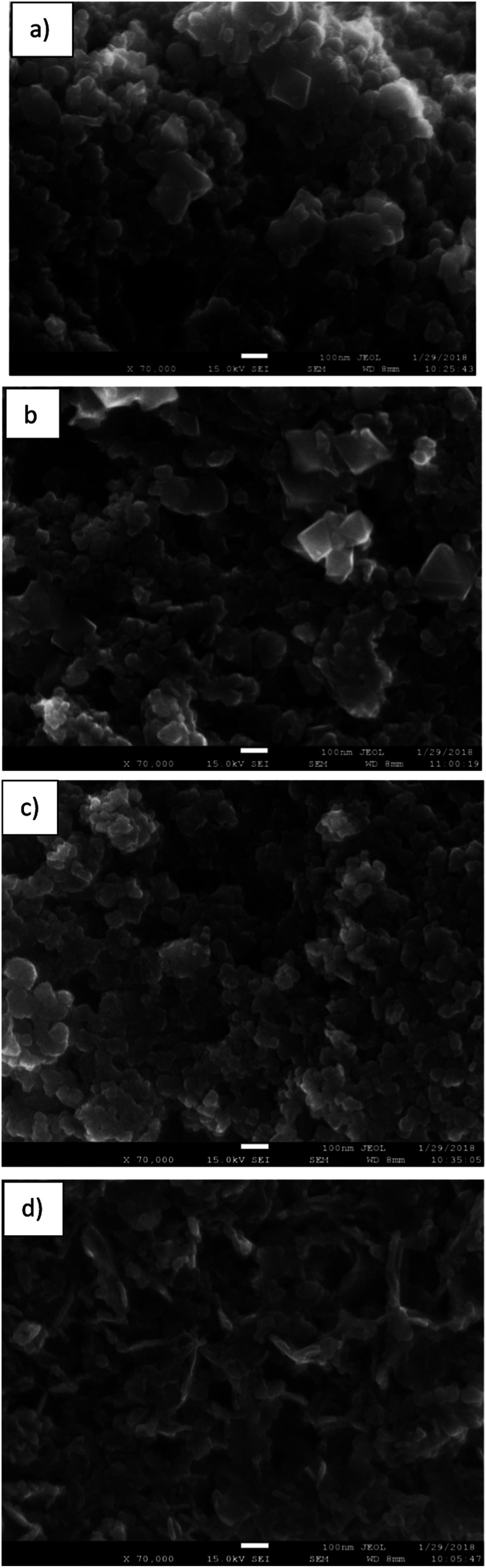
SEM images of the binary metallic oxides synthesized: (a) BMO1; (b) BMO2; (c) BMO3 and (d) BMO4.

Fig. 4 shows SEM images of the different BMOs. All reveal agglomerated particles of near-spherical morphology, similar to those reported for other samples of similar composition.^46^ No changes in this morphology are observed with the variation in the Zn/Mn content. Tetragonal crystals characteristic of the spinel phase are also visible (parts a and b of this figure).

**Fig. 5:**
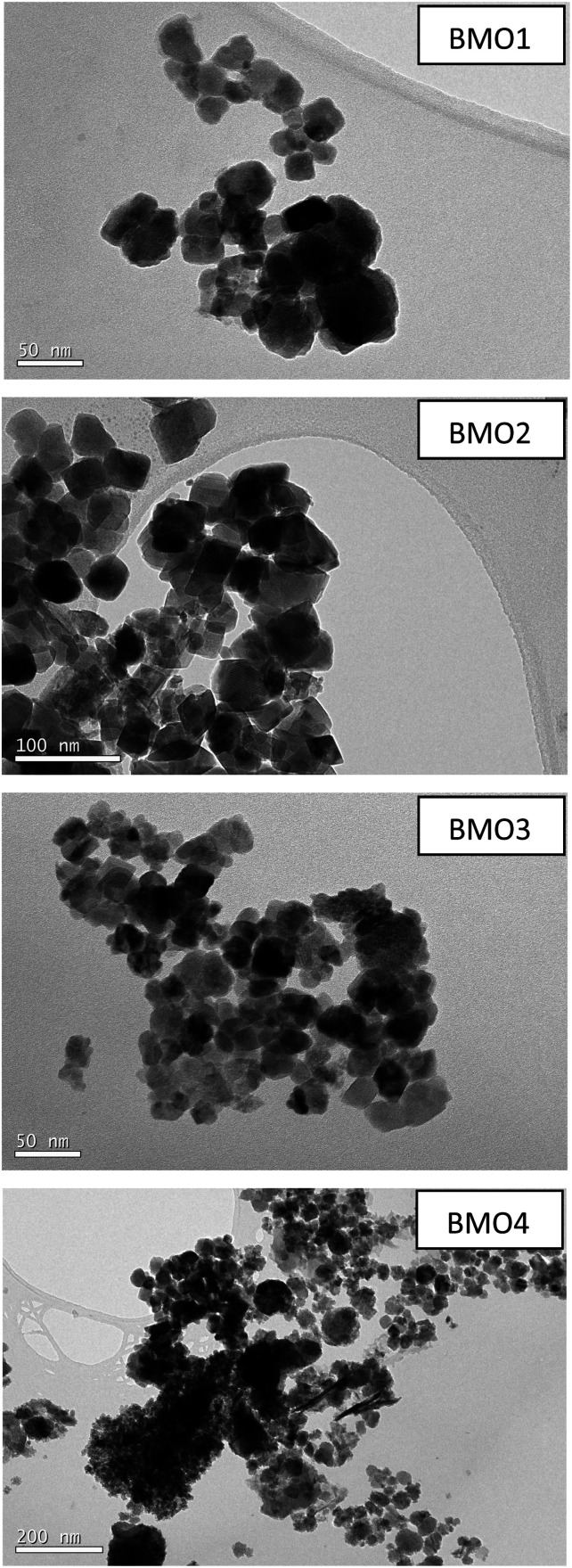
TEM images of the binary metallic oxides synthesized.

Fig. 5 shows TEM images of the different BMOs. Two different morphologies are present. In BMO1, BMO2 and BMO3 samples, spherical particles are mainly observed, while in BMO4 both spherical and hexagonal-shaped particles are visible. The hexagonal shaped particles could be related to the higher content of ZnO particles^21^ in agreement with our results of the Rietveld analysis (see Table 3). The mean diameter of the particles is 40, 50 and 35 nm for BMO1, BMO2 and BMO3 respectively. An increase in the Mn content leads to an increase in particle size, as previously reported.^43^

**Fig. 6:**
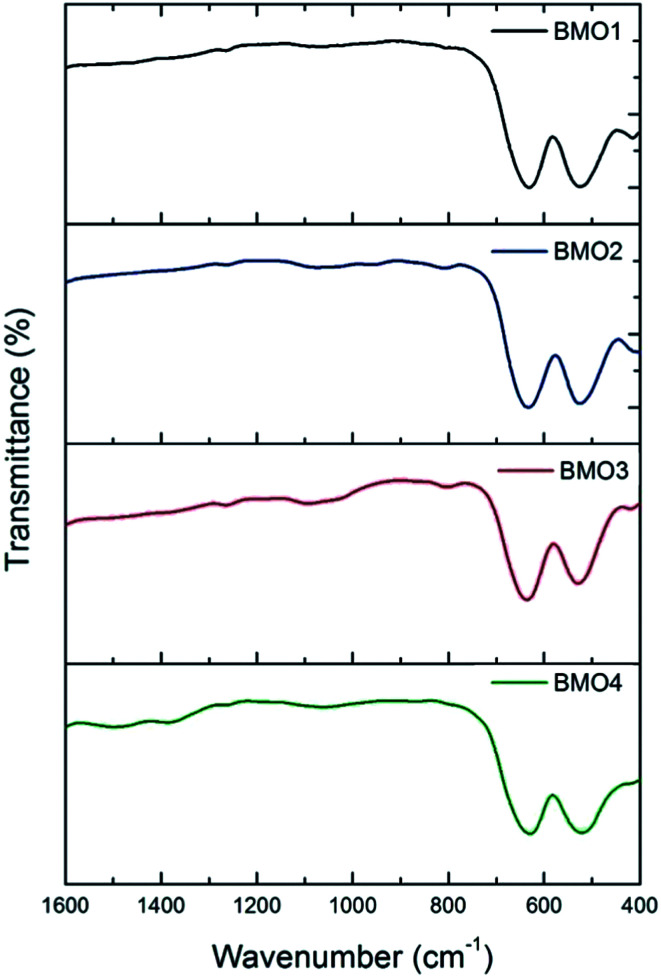
FTIR spectra of the binary metallic oxides synthesized.

Fig. 6 shows the FTIR spectra for the BMOs. All show two main absorption bands at approximately 527 and 634 cm^−1^. These may be respectively attributed to distortion vibration of Mn^3+^ cations in an octahedral environment, and stretching vibration at Mn–O tetrahedral sites. A weaker band is also observed at around 420 cm^−1^, due to stretching vibration of Mn^3+^ at octahedral sites.^44–46^

**Fig. 7:**
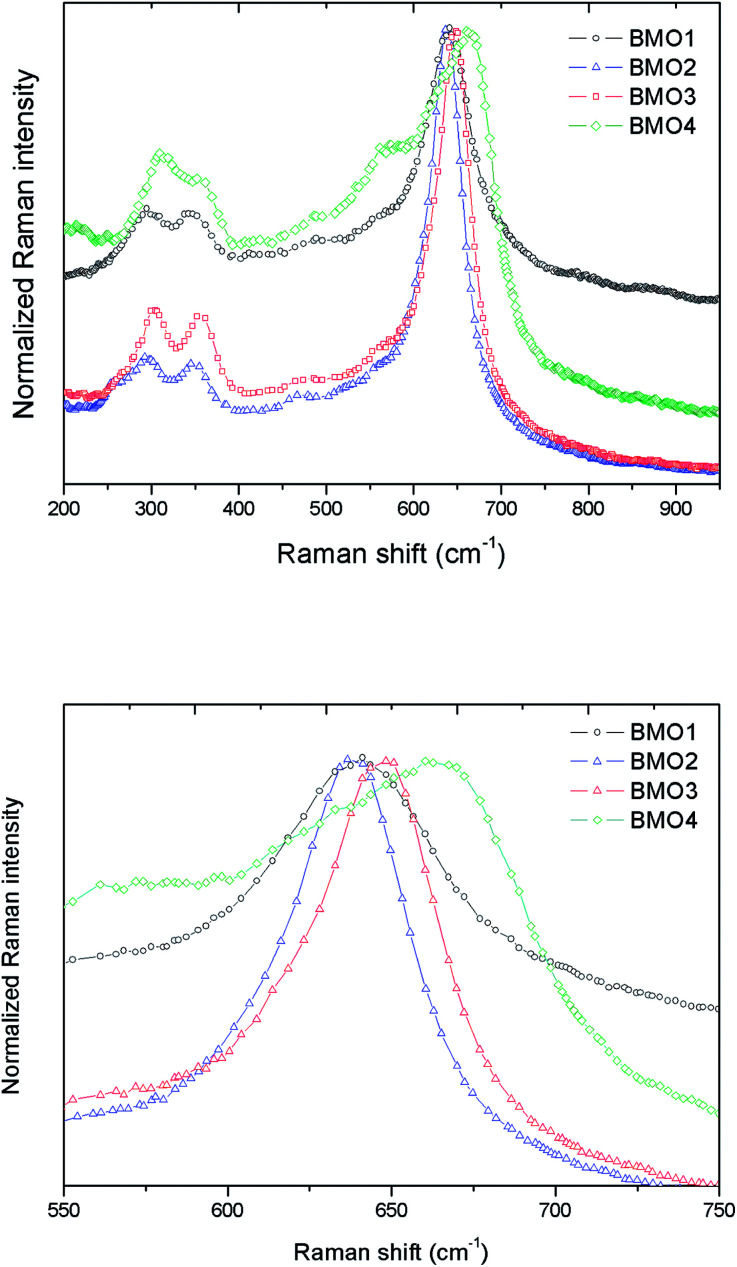
(a) Normalized Raman spectra for different Zn/Mn ratios, and (b) zoom of the Raman peak in the 645–670 cm^−1^ range.

Fig. 7a shows the normalized Raman spectra for the different Zn_*x*_Mn_3−*x*_O_4_ samples. In all the spectra the bands are attributable to ZnMn_2_O_4_. These results are consistent with those obtained in the XRD analysis.

According to the group theory, oxides of the stoichiometry XMn_2_O_4_ (where X = Zn, Mn) with a spinel-type crystalline structure and *I*4_1_/*amd* space group have 10 active Raman modes: Γ = 2A_1g_ + 3B_1g_ + B_2g_ + 4E_g_.^47^ However, the present spectra do not show all these modes; indeed, the maximum number ever observed has been seven.^48,49^ The vibration modes above 600 cm^−1^, correspond to the movement of oxygen atoms in the tetrahedral AO_4_ groups, and reflect an A_1g_ symmetry.

The low frequency modes are characteristic of octahedral sites (BO_6_). Other authors report a high variation in the position of the Raman peaks for ZnMn_2_O_4_. Malavasi *et al.*^48^ reported peaks at 300.2, 320.5, 381.9, 475.5 and 677.6 cm^−1^, Tortosa *et al.*^49^ at 276, 316, 320, 369, 463, 563, 630 and 663 cm^−1^, and Li *et al.*^50^ at 171, 321, 384, 476, 635 and 677 cm^−1^. In the present work, these peaks appear around 301, 350, 477 and 648 cm^−1^. The band in the 645–670 cm^−1^ range might be attributed to manganese oxides and Zn/Mn complexes.^47^Fig. 7b shows a zoom of this Raman peak. Note that as the Mn content increases, the height of the peak decreases. According to XPS, in the sample with the higher Mn content, the predominant oxidation state is Mn^2+^, hence the differences observed in the Raman peak could be explained by the substitution of Zn^2+^ by Mn^2+^ (which is larger) in the BMOs,^51^ which would lead to a reduction in the bond distances. In addition, a broadening of the Raman bands can be appreciated related to the purity and crystallinity of the samples. These results are in good agreement with those obtained from the Rietveld refinement where highest percentage of impurities leading to a broader Raman bands.

### Magnetic properties

**Fig. 8:**
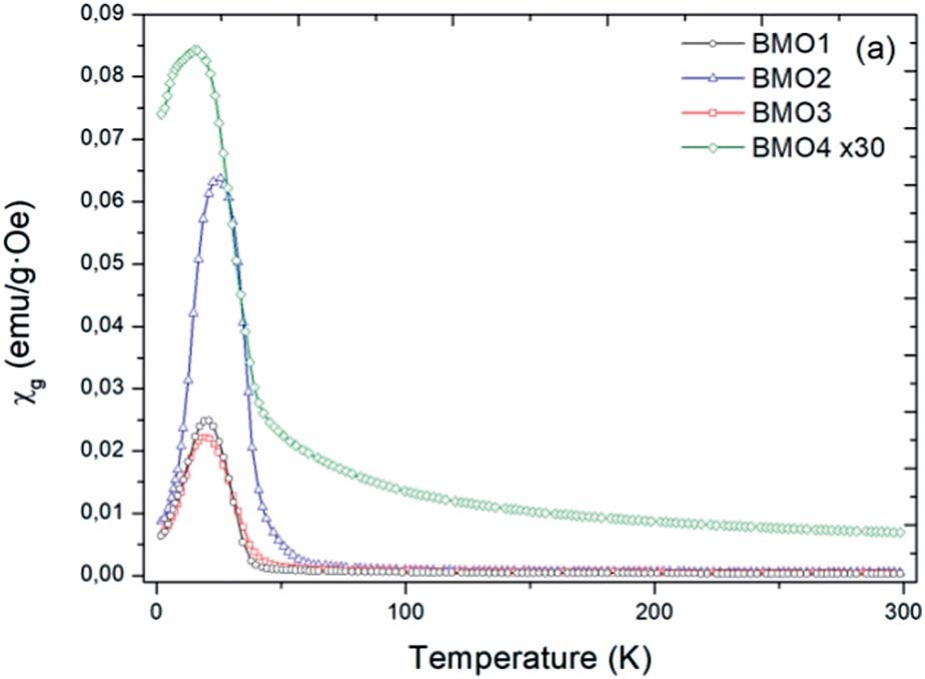
Magnetic susceptibility per unit mass *versus* temperature in a field of *H* = 1000 Oe.

**Fig. 9:**
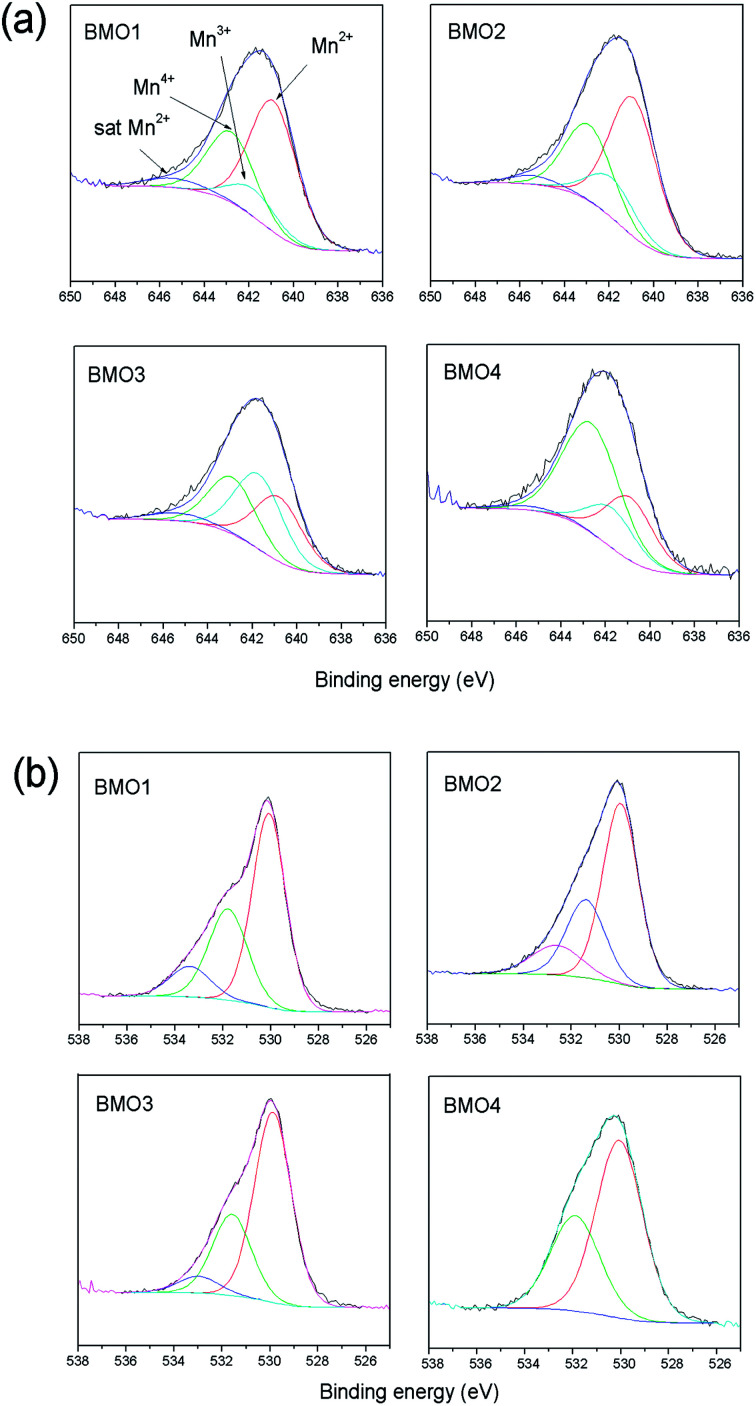
(a) XPS Mn 2p spectra and (b) XPS O 1s spectra of the binary metallic oxides.

Fig. 8 shows the variation in magnetic susceptibility per unit mass (*χ*_g_) *vs.* temperature over the range 2–300 K in a 1000 Oe field. All the BMOs behave in a similar way, the magnetic susceptibility rises to a peak value and then decreases. These inflection points allow us to determine the Curie temperature (*T*_c_) for each BMO (Table 5). As the value of *x* increases in the nominal composition of the Zn_*x*_Mn_3−*x*_O_4_ phases, the *T*_c_ reduces, as reported by Nádherný *et al.*^52^ BMO2 shows the greatest magnetic susceptibility, in agreement with the XPS results (Fig. 9). For this sample, the majority charge state for Mn ions is +2, hence, since Mn^2+^ has the largest magnetic dipole moment (5.9μB), a largest magnetic moment and saturation magnetization are expected.

**Table 5:** Curie temperatures (*T*_N_), magnetization at saturation (*M*_s_) and coercive field (*H*_c_) for the different BMO studied

Sample	*T*_N_ (K)	*M*_s_ (emu g^−1^)	*H*_c_ (Oe)
BMO1	24.1	6.17	1720
BMO2	25.5	13.19	1720
BMO3	19.7	5.86	1220
BMO4	15.5	1.56	940

Different theories exist regarding the magnetic properties of (Zn, TM) O-type (with transition metals) oxides. Sharma *et al.*^53^ and Kundaliya *et al.*^54^ explain the ferromagnetism of these materials by assuming a uniform distribution of Mn in a ZnO matrix, or through the existence of phases with a Zn_*x*_Mn_2−*x*_O_3_ stoichiometry stabilized by oxygen vacancies. Others explain it *via* the coexistence of Mn atoms in different states of oxidation.^55^Fig. 9 shows the XPS spectra for the 2p Mn structure of each BMO. The bond energies for Mn 2p3/2 of MnO, Mn_2_O_3_ and MnO_2_ fall within the ranges 640.6–641.7, 641.7–641.9 and 641.9–642.6 eV respectively.^56,57^Table 6 shows the elemental atomic percentages for the different Zn/Mn ratios, and the percentages according to the Mn oxidation state. In all the examined BMOs, mixtures of Mn^2+^, Mn^3+^ and Mn^4+^ are obtained. The XPS spectra for the O 1s are also shown in Fig. 9. In all cases, the obtained spectra exhibit asymmetric peaks. In this respect, there is a controversy in the interpretation of O 1s XPS spectra for different oxides. The band peaked at around 534 eV in some cases is attributable to hydroxyl groups OH, or other radicals on the sample surface^58,59^ or oxygen vacancies.^60,61^ For the present BMOs, none of these possibilities can be ruled out. Either, the existence of oxygen vacancies created to compensate the electric charge or the different Mn ions could be equally responsible for the magnetic behavior.

**Table 6:** Atomic ratio Mn/Zn calculated from the data obtained by XPS and distribution of the percentage of Mn according to the oxidation state

Sample	Atomic ratio Mn/Zn	Mn^2+^	Mn^3+^	Mn^4+^
BMO1	8.3	14.5	3	7.8
BMO2	12.2	15.4	4.4	8.5
BMO3	1.3	6.3	7.3	5.5
BMO4	0.6	3.4	2.1	7.1

**Fig. 10:**
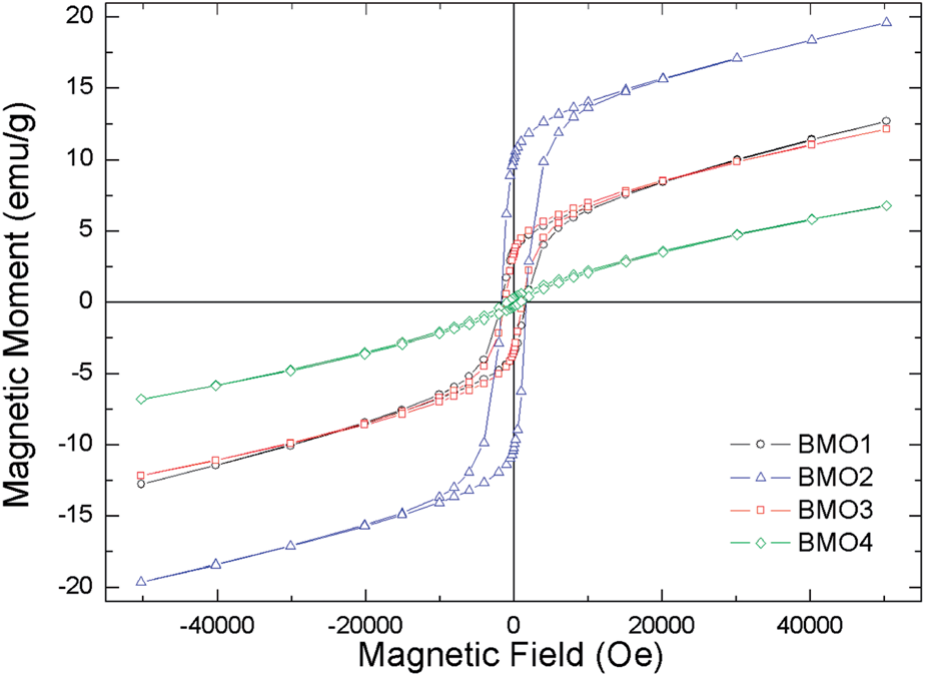
Magnetization curves in the applied magnetic field at a temperature of 10 K.

Fig. 10 shows the magnetization curves *versus* applied field. All are representative of a ferromagnetic material with magnetic domains. As expected, the magnetic saturation (*M*_s_) increased with the Mn content, reaching a value of 13.2 emu g^−1^ for BMO2. Table 5 shows the different *M*_s_ values obtained for the BMOs in a coercive field (*H*_c_). *M*_s_ decreases with increasing Zn content, a consequence of the presence of its non-magnetic cations.^52^ This was particularly clear for BMO4, which has the lowest Zn/Mn ratio and Mn mainly in the Mn^4+^ oxidation state (as determined by XPS). Thus – in agreement with other authors who have studied spinel BMOs of Zn_*x*_Mn_3−*x*_O_4_ stoichiometry^52^ – the magnetic properties of the present BMOs can be explained according to a model of nanometric-scale ferrimagnetic clusters of Mn_3_O_4_ in an antiferromagnetic ZnMn_2_O_4_ matrix.^62,63^ These results are in good agreement with previous studies on Zn_*x*_Mn_3−*x*_O_4_ phases formed by members of Mn_3_O_4_ and ZnMn_2_O_4_. The Mn_3_O_4_ phase contains Mn in oxidation state II at tetrahedral positions, and in state III at octahedral positions. As the Zn content increases (with increasing *x* in the stoichiometry Zn_*x*_Mn_3−*x*_O_4_), the tetrahedral sublattice occupied by Mn^2+^ begins to be occupied by Zn^2+^. Table 6 shows how, in most cases, an increase in *x* is accompanied by a reduction in Mn^2+^ and an increase in Mn^3+^. The existence of Mn^4+^ can be explained by the presence, in some samples, of MnO_2_-type impurities (detected during Rietveld refinement analysis) or oxygen vacancies, as suggested by Kundaliya *et al.*^54^

## Conclusions

The present work reports the synthesis and characterization of different Zn/Mn BMOs from the black mass of spent Zn/C and alkaline batteries. The XRD patterns of most of these BMOs show diffractions maxima attributable to tetragonal symmetry s.g. *I*4_1_/*amd* and a spinel-type type structure. The Rietveld refinement results reveal phases of Zn_*x*_Mn_3−*x*_O_4_ stoichiometry with magnetic properties explainable *via* a model of nanoscale clusters of ferrimagnetic Mn_3_O_4_ in an antiferromagnetic ZnMn_2_O_4_ matrix. All the BMOs contain Mn^2+^, Mn^3+^ and Mn^4+^. The synthesis described provides a simple method to obtain Zn_*x*_Mn_3−*x*_O_4_compounds useful potential for several applications. This process would appear to be a good way to obtain spinel from spent batteries – an useful product from a dangerous waste.

## Conflicts of interest

The authors declare no conflict of interest.

## Supplementary Material
